# Intercomparison of the ITS-90 Radiance Temperature Scales of the National Physical Laboratory (U.K.) and the National Institute of Standards and Technology

**DOI:** 10.6028/jres.099.065

**Published:** 1994

**Authors:** G. Machin, B. Carol Johnson, C. Gibson, R. L. Rusby

**Affiliations:** The National Physical Laboratory, Queens Road, Teddington, Middlesex, TW11 OLW, United Kingdom; National Institute of Standards and Technology, Gaithersburg, MD 20899-0001, USA; The National Physical Laboratory, Queens Road, Teddington, Middlesex, TW11 OLW, United Kingdom

**Keywords:** intercomparison, ITS-90, radiance temperature, temperature scales

## Abstract

An intercomparison of radiance temperature scales has been performed by the National Physical Laboratory (NPL) and the National Institute of Standards and Technology (NIST) using a standard transfer pyrometer operating at a wavelength of approximately 1000 nm. It was found that the radiance temperature scales established by the two laboratories were in agreement to 0.1% or better of the temperature over the range 1000 °C to 2500 °C.

## 1. Introduction

To maintain confidence in national standards it is essential to periodically inter-compare them to confirm their equivalence. The last intercomparison of radiance temperature scales between the National Physical Laboratory (NPL) and the National Bureau of Standards (NBS) (now NIST) was undertaken in the early 1970s [1] using high stability tungsten ribbon lamps calibrated, in turn, at the two laboratories. Subsequently, the results of this intercomparison were incorporated in a Memorandum of Understanding (MoU) between the two laboratories on the equivalence of their respective realizations of the International Practical Temperature Scale of 1968 (IPTS-68). According to the MoU the scales, established by NPL and NBS on the basis of the lamps at 660 nm, were in agreement at the 2 standard deviation level to within 0.6 °C from 1000 °C to 1500 °C and to within 2 °C from 1500 *°*C to 2200 °C. Note that expanded uncertainties are used in this paper, corresponding to a 95% level of confidence assuming a normal distribution [2].

Since the early 1970s both NPL and NIST have made significant changes to the way they establish their radiance temperature scales. In particular, both laboratories have developed facilities to make radiance temperature intercomparisons in the infra-red using blackbody sources. Moreover, a new temperature scale, the ITS-90, has been introduced [3]. In view of these changes and because most commercial radiation thermometers now operate at infra-red wavelengths (rather than at 660 nm) it was decided to perform an entirely new intercomparison of radiance temperature scales at a longer wavelength.

## 2. Radiance Temperature Scales

### 2.1 The NPL Radiance Temperature Scale

The radiance temperature scale at NPL is maintained on the basis of two high stability evacuated tungsten ribbon lamps. These are described by Quinn [4] and Coates [5]. NPL realizes the ITS-90 using a blackbody cavity (with an emissivity of > 0.99995) immersed in a substantial ingot (0.8 kg) of high purity gold. The radiance of the gold point (1337.33 K) is measured using the NPL primary pyrometer at 655 nm. This provides the fiducial point for the scale. The two lamps are calibrated using a “bootstrap” technique. The current through each lamp ribbon is adjusted until the radiance is about that of the gold point. One lamp (lamp 1) is held at this point while the radiance of the second (lamp 2) is set at double the radiance of lamp 1. The temperature of lamp 2 is then calculated and thereafter the radiance of lamp 1 is increased to that of lamp 2. This cycle is repeated up to the maximum operating temperature of the lamps (1700 °C) and the scale is thus established. Other evacuated lamps are calibrated up to 1700° C relative to one of these lamps. Gas filled high stability tungsten ribbon lamps are calibrated up to 2200 °C while for higher temperatures (up to 2650 °C) blackbody lamps are used.

Lamps are useful as stable transfer standard sources, but are not suitable for the calibration of many modern radiation thermometers. This is because these thermometers generally operate at longer wavelengths (e.g., 0.9 μm) and have fields of view that are typically larger than the width of a tungsten ribbon (1.5 mm or 3 mm). Recently NPL has acquired a variable temperature blackbody for the calibration of such thermometers. This device is based on the design of Groll and Neuer [6]. It has an overall temperature range of 1000 °C to 2650 °C and an aperture of 15 mm. The temperature of the blackbody is assigned using an IKE-Linearpyrometer (the LP2)^1^. This instrument is described by Schreiber, Neuer, and Wörner [7]. It is calibrated at its operating wavelengths (650 nm, 804 nm, and 906 nm) against the gold point blackbody. It can then be used to measure the temperature of the variable temperature blackbody (and other sources). Its calibration was verified at 650 nm by using it to measure the radiance temperature of calibrated standard lamps (up to 2200 °C). The temperature of these lamps as measured by the LP2, over the range 1000 °C to 2200 °C, departed by less than ±0.5 °C from their primary calibration, well within their calibration uncertainty.

### 2.2 The NIST Radiance Temperature Scale

The radiance scale as realized by NIST is described in detail by Mielenz et al. [8]. It is generated from the gold point, as at NPL. The NIST gold point blackbody is used to assign a temperature to a Quinn and Lee [9] tungsten ribbon cylindrical envelope lamp held at 1255.64 °C. This transfer is performed using the NIST primary photoelectric pyrometer which operates at a wavelength of 655.7 nm. The temperature scale is then transferred to a variable temperature blackbody (manufactured by Thermogage, Inc.) using the photoelectric pyrometer. The blackbody has an aperture of 25 mm and a maximum operating temperature of approximately 2700 °C. The temperature of the cavity is derived from radiance ratio measurements at 655.7 nm, using the NIST primary photoelectric pyrometer and the lamp at 1255.64 °C. This temperature is approximately eight times the spectral radiance of the gold point blackbody at 655.7 nm. As most practical radiation thermometers operate at a wavelength significantly different from 655.7 nm a correction must be applied to the radiance temperature of the variable temperature blackbody as obtained by the NIST primary photoelectric pyrometer. This is because the spectral radiance temperature is dependent upon wavelength, emissivity (0.995 for the NIST blackbody) and thermodynamic temperature.

Since the starting point of both the NIST and the NPL scales is the freezing point of gold (ITS-90 temperature 1337.33 K) the radiance temperature scales should be identical within the calibration and measurement uncertainties.

Note that neither at NPL nor NIST is the temperature scale maintained on the variable temperature blackbody sources. Their temperatures must be determined at each point by measurement with the LP2 (at NPL) or relative to the Quinn and Lee ribbon lamp (at NIST).

## 3. The Intercomparison

The intercomparison was performed using a Standard Transfer Pyrometer (STP). This was a Land IR Ltd, Cyclops 52 radiation thermometer modified to meet NPL specifications. Its electronics were re-designed to improve the resolution and stability and provide a voltage output proportional to the radiance. A sensor measures the temperature of the silicon photodiode and the instrument automatically compensates for any changes from this effect. An additional lens was fitted to the front of the STP to reduce the field of view (f.o.v.) to 2.8 mm at 62 cm. The radiation thermometer operates at a wavelength of approximately 1000 nm.

The STP was calibrated at NPL using the variable temperature blackbody. A temperature was assigned to the blackbody cavity using the LP2. This was done at 906 nm but investigation showed that the temperature of the blackbody radiator was essentially the same whether it was measured at 650 nm, 804 nm, or 906 nm.

At each blackbody controller setting between eight and twelve individual readings were taken and the mean of these results was used to ascribe a temperature, *T*, to the radiator. The voltage output, *V*, of the STP for that temperature was determined using a calibrated digital voltmeter. The repeatability within each measurement set was between ±0.2°C; when measurements were spaced over several days and the calibration set-up was realigned, the repeatability was somewhat larger but still less than 0.35 °C.

The calibration was performed over the range 1000 °C to 2000 °C. As the STP operates at about 1000 nm and the emissivity of the blackbody is considerably greater than 0.995, a negligible uncertainty (<0.25°C at 2500 °C) is introduced by this transfer. Residual temperature gradients in the blackbody were accommodated by de-focusing the LP2 slightly (by moving the LP2 backwards) so that its field of view approximately matched that of the STP. Since both the LP2 and the STP were then sampling the same area of the blackbody aperture the effect of any temperature gradients were, to first order, compensated for. The measured radiance did not vary by more than the equivalent of 1 °C over the 4 mm central region of the blackbody aperture.

**Fig. 1:**
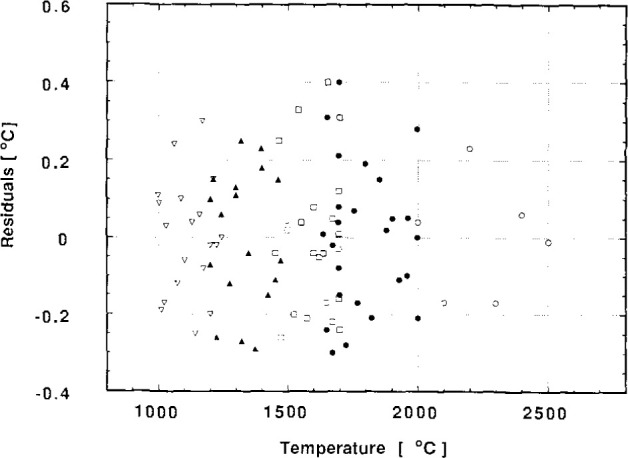
The residuals, expressed in terms of temperature, for the fit of the polynomials representing the original and subsequent NPL calibration of the NPL Standard Transfer Pyrometer (STP). Different symbols represent different overlapping subranges, these are; ∇ 1000 °C to 1243 °C, ▲ 1200 °C to 1470 °C, □ 1450 °C to 1700 °C, ● 1636 °C to 2000 °C, and ○ 2000 °C to 2500 °C. The radiance temperature of the NPL blackbody source was determined through the calibration procedure described in Sec. 2.1 of the text.

The results of the calibration were fitted by polynomials *V* vs *T* so that the temperature could be easily calculated from a measured STP output voltage. The entire range of the instrument was covered by separate polynomials fitted to the data for each of four overlapping subranges. These subranges were 1000 °C to 1250 °C, 1200 °C to 1475 °C, 1450 °C to 1700 °C, and 1635 °C to 2000 °C. The residuals of the fits are shown in Fig. 1. The residuals are scattered evenly and most lie within 0.3 °C of zero. The standard deviation of the residuals was used to assign a value of 0.3 °C for the expanded uncertainty of the whole of the calibration and fitting procedure. This value has been incorporated in the overall uncertainty given in the next section.

At NIST the STP was recalibrated using the variable temperature blackbody source. The temperature of the blackbody was determined by the technique described in Sec. 2.2. The blackbody was then moved so that it was viewed by the STP. The STP output was then measured. This procedure was repeated at each calibration temperature, and all the temperatures were repeated at least once on a different day. Again each calibration result was the mean of several individual readings. The expanded uncertainty for these measurements also appeared to be about 0.3 °C.

While at NIST it was decided to extend the range of the intercomparison beyond 2000 °C, to 2500 °C. This was done by fitting a neutral filter of 10% transmission to the front of the STP for temperatures in excess of 2000 °C.

On return to NPL the instrument was recalibrated, including the range above 2000 °C. An extra polynomial was fitted to the data from 2000 °C to 2500 °C and the residuals of the fit can be seen in Fig. 1.

## 4. The Results of the Intercomparison

**Fig. 2:**
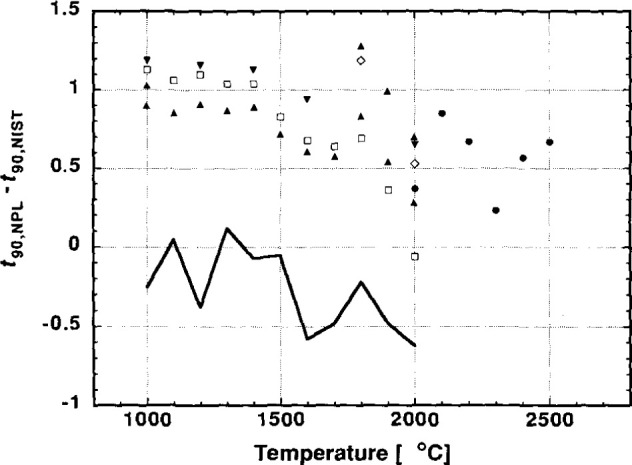
Differences between the calibration of the NPL Standard Transfer Pyrometer (STP) obtained at NPL, *t*_90_,_NPL_, and NIST, *t*_90_,_NIST_. The radiance temperature of the NIST variable blackbody source was determined through the procedure described in Sec. 2.2 of the text. Different symbols represent different calibration runs at NIST, these are: ▲ 05 May 92, ▼ 08 May 92, ◊ 11 May 92, and □ 15 May 92. The solid circles represent the extension of the intercomparison to 2500 °C. The solid curve represents the differences between the calibration of the STP at NPL before and after the work at NIST.

The results of the intercomparison are shown in Table 1 and Fig. 2. In Table 1, column 1 is the nominal intercomparison temperature in degrees Celsius. Column 2 is the difference between the NPL and the NIST calibrations of the STP. Column 3 is the expanded uncertainty that NIST ascribe to the radiance temperature of the variable temperature blackbody at 1000 nm. See Waters et al. [10] for a description of how uncertainties in this type of scale realization are determined. We have added a small contribution caused by the uncertainty in the effective wavelength. NIST usually also includes the uncertainty in the absolute value of the gold point temperature (see Mielenz, Saunders, and Shumaker [11] for details). But as the assigned ITS-90 value has been used in both laboratories this uncertainty has not been included. Column 4 is the expanded uncertainty of the calibration of the NPL blackbody with the LP2 as validated using the lamp (see Sec. 2.1) and column 5 gives the combined NIST and NPL uncertainties.

In the short term, the results at NPL and NIST show that the STP was repeatable to ±0.3 °C. The medium term repeatability is assessed from the difference between the NPL calibrations of the STP before and after the NIST visit. This difference is shown as the solid curve on Fig. 2. Unfortunately, because the STP developed a fault requiring repair shortly after return to NPL, only one recalibration could be performed. It is therefore important not to place too much weight on these measurements as it was not possible to repeat them. Column 5 of Table 1 gives the combined expanded uncertainty of the intercomparison between NIST and NPL, from columns 3 and 4, and includes the short term reproducibility of the STP.

## 5. Discussion

The NPL and the NIST infrared radiance temperature scales are in agreement to 0.1% of the temperature, the agreement improving as the temperature increases. They are, by and large, consistent with each other to within the 95% confidence intervals that are ascribed by the two laboratories.

It is surprising, however, that the largest differences occurred at the lower temperatures, close to the gold point reference temperature. To check the results at NIST the variable temperature blackbody was calibrated at 1064 °C directly against the gold point, independent of the lamp. This gave the same results as shown in Table 1.

A more likely explanation for the discrepancy lies in the use of the STP. First, its medium term repeatability could be responsible for part of the difference. Second, the f.o.v. of the STP matches neither that of the NIST primary pyrometer nor the LP2, therefore any temperature gradients were imperfectly accounted for. This could lead to an uncertainty in the calibration of the STP. As mentioned earlier, NPL de-focus the LP2 to better match the f.o.v. of the STP with the laboratory pyrometer – but effects due to imperfect matching and alignment could remain.

This intercomparison differs in some important respects from that performed by Lee et al. [1]. The main differences are that this intercomparison was detector based rather than source based – in itself something of a novelty since detectors with adequate stability and range are a comparatively recent innovation. The actual sources used in the intercomparison were blackbodies rather than lamps and the intercomparison was performed at long wavelengths, 900 nm to 1000 nm, rather than the more traditional 660 nm. Also in this instance only one artifact was exchanged. However, its prime aim was to confirm that the NIST and NPL radiance temperature scales are still in good agreement, despite the many changes that have taken place at the two laboratories, and this has been achieved, within the limits given above.

In view of the factors above, it is pleasing to note that the observed temperature differences above 1500 *°C* lie well within the 2 *°C* band allowed by the original MoU. This was found to be the case up to 2500 °C and the MoU should be extended (currently limited to 2200 °C) to reflect this new data. However below 1500 °C the MoU does not fully cover the difference observed. This is largely because, in switching to variable temperature blackbodies to provide standards of increased flexibility appropriate to modern industrial needs, both NIST and NPL have had to accommodate slight increases in the source temperature uncertainty. In the absence of a new intercomparison of lamps at 660 nm the revised MoU should relate to the present results, covering infrared wavelengths.

Finally the fact that some of the differences at lower temperatures lie outside the combined expanded uncertainties reinforces the fact that great care must be exercised when radiation thermometers are calibrated against blackbody sources. In particular, the requirements that the source aperture significantly overfills the instrument’s f.o.v. and emits uniform radiance must be met to ensure that a good calibration can be performed.

## 6. Conclusion

These results show that the infrared temperature scales as established at NPL and NIST are equivalent to each other at or better than the 0.1% of the temperature. The original MoU needs some modification to take into account the larger than expected differences found at lower temperatures but it can be extended upward in range from 2200 °C to 2500 °C.

## Figures and Tables

**Table 1:** The results of the 1992 intercomparison of the NIST and NPL radiance temperature scales measured with the NPL Standard Transfer Pyrometer (STP) operating at approximately 1000 nm

Nominal temperature (°C)	*t*_90,NPL_–*t*_90,NIST_ (°C)	NIST 2*σ* uncertainty (°C)	NPL 2*σ* uncertainty (°C)	Combined 2*σ* uncertainty^a^ (°C)
1000	+1.06	0.5	0.7	0.9
1100	+0.96	0.6	0.7	1.0
1200	+1.06	0.6	0.8	1.1
1300	+0.96	0.7	1.0	1.2
1400	+1.02	0.7	1.0	1.3
1500	+0.77	0.8	1.1	1.4
1600	+0.74	0.9	1.2	1.5
1700	+0.61	1.0	1.3	1.7
1800	+1.03	1.1	1.4	1.8
1900	+0.63	1.3	1.6	2.1
2000	+0.36	1.4	1.7	2.2
2100	+0.85	1.5	1.9	2.5
2200	+0.67	1.7	2.0	2.7
2300	+0.23	1.9	2.3	3,0
2400	+0.57	2.1	2.5	3.3
2500	+0.67	2.3	2.7	3.6

a Includes uncertainty in the calibration of the STP.

## Footnotes

1 Certain commercial equipment, instruments, or materials arc identified in this paper to foster understanding. Such identification does not imply recommendation or endorsement by the National Institute of Standards and Technology or the National Physical Laboratory, nor does it imply that the materials or equipment identified arc necessarily the best available for the purpose.
